# Supplementary material for: Evaluating implementation research outcomes for a task-sharing mental health intervention: A systematic review of the Friendship Bench
Source: Glob Ment Health (Camb). 2025 Jun 11;12:e65. doi: 10.1017/gmh.2025.10025 (PMC12231309; doi:10.1017/gmh.2025.10025)
Supplement: Patena et al. supplementary material 2 — Patena et al. supplementary material [file S2054425125100253sup002.docx]

**Appendix 2 – Search strategy**

(mental health OR mental illness OR mental disorder OR common mental health disorder OR common mental disorders OR depression OR generalized anxiety disorder OR panic disorder OR phobias OR social anxiety disorder OR obsessive-compulsive disorder OR post-traumatic stress disorder [tw]) AND “Friendship Bench” AND (acceptability OR patient acceptance OR patient compliance OR adherence OR patient participation OR diffusion of innovation OR healthcare utilization OR adoption OR uptake OR empirically supported treatment OR appropriateness OR program evaluation OR program effectiveness OR benchmarking OR cost OR economics OR feasibility OR outcomes assessment OR process assessment OR scale OR measure OR fidelity OR integrity OR penetration OR sustainability OR evidence-based intervention OR evidence-based practice OR intervention) AND (lmic OR low income country OR low income countries OR middle income country OR middle income countries OR global south OR developing country OR developing countries OR medically underserved area OR medically underserved areas OR resource poor OR low resource OR third world country OR third world countries OR less developed country OR less developed countries OR least developed country OR least developed countries OR africa OR central asia OR western asia OR southeastern asia OR indian ocean islands OR central america OR south america OR eastern europe OR transcaucasia OR caribbean region OR pacific islands OR afghanistan OR afghan OR burkina faso OR burkinabe OR burundi OR burundian OR central african republic OR central african OR chad OR chadian OR congo OR congolese OR eritrea OR eritrean OR ethiopia OR ethiopian OR gambia OR gambian OR guinea OR guinean OR guinea bissau OR bissau guinean OR korea OR korean OR liberia OR liberian OR madagascar OR malagasy OR malawi OR malawian OR mali OR malian OR mozambique OR mozambican OR niger OR nigerien OR rwanda OR rwandan OR sierra leone OR sierra leonean OR somalia OR somali OR sudan OR sudanese OR syria OR syrian OR togo OR togolese OR uganda OR ugandan OR yemen OR yemeni OR zambia OR zambian OR angola OR angolan OR algeria OR algerian OR bangladesh OR bangladeshi OR benin OR beninese OR bhutan OR bhutanese OR bolivia OR bolivian OR cape verde OR cape verdeans OR cambodia OR cambodian OR cameroon OR cameroonian OR comoros OR comorian OR cote d'ivoire OR ivory coast OR ivorian OR djibouti OR djiboutian OR egypt OR egyptian OR el salvador OR salvadoran OR eswatini OR emaswati OR swaziland OR swazi OR ghana OR ghanaian OR haiti OR haitian OR honduras OR honduran OR india OR indian OR indonesia OR indonesian OR iran OR iranian OR kenya OR kenyan OR kiribati OR I-kiribati OR kyrgyzstan OR kyrgyz OR laos OR lao OR laotian OR lebanon OR lebanese OR lesotho OR basotho OR mauritania OR mauritanian OR micronesia OR micronesian OR mongolia OR mongolian OR morocco OR moroccan OR myanmar OR burmese OR nepal OR nepalese OR nicaragua OR nicaraguan OR nigeria OR nigerian OR pakistan OR pakistani OR papua new guinea OR papua new guinean OR philippines OR filipino OR samoa OR samoan OR sao tome principe OR santomean OR senegal OR senegalese OR solomon islands OR solomon islander OR sri lanka OR sri lankan OR tanzania OR tanzanian OR tajikistan OR tajik OR tajikistani OR east timor OR timor leste OR timorese OR tunisia OR tunisian OR ukraine OR ukrainian OR uzbekistan OR uzbek OR vanuatu OR vanuatuan OR vietnam OR vietnamese OR west bank OR gaza OR zimbabwe OR zimbabwean OR albania OR albanian OR american samoa OR american samoan OR argentina OR argentinian OR armenia OR armenian OR azerbaijan OR azerbaijani OR belarus OR belarusian OR belize OR belizean OR bosnia OR herzegovina OR bosnian OR botswana OR batswana OR brazil OR brazilian OR bulgaria OR bulgarian OR china OR chinese OR colombia OR colombian OR costa rica OR costa rican OR cuba OR cuban OR dominica OR dominican OR dominican republic OR equatorial guinea OR equatoguinean OR ecuador OR ecuadorean OR fiji OR fijian OR gabon OR gabonese OR georgia OR georgian OR grenada OR grenadian OR guatemala OR guatemalan OR guyana OR guyanese OR iraq OR iraqi OR jamaica OR jamaican OR jordan OR jordanian OR kazakhstan OR kazakhstani OR kosovo OR kosovar OR libya OR libyan OR lithuania OR lithuanian OR malaysia OR malaysian OR maldives OR maldivian OR marshall islands OR marshallese OR mauritius OR mauritian OR mexico OR mexican OR moldova OR moldovan OR montenegro OR montenegrin OR namibia OR namibian OR macedonia OR macedonian OR palau OR palauan OR panama OR panamanian OR paraguay OR paraguayan OR peru OR peruvian OR romania OR romanian OR russia OR russian OR serbia OR serbian OR south africa OR south african OR saint lucia OR saint lucian OR saint kitts OR saint vincent OR grenadines OR saint vincentian OR grenadinian OR seychelles OR seychellois OR suriname OR surinamese OR thailand OR thai OR tonga OR tongan OR turkey OR turkish OR turkmenistan OR turkmen OR tuvalu OR tuvaluans OR venezuela OR venezuelan)
